# Addendum for Euro Surveill. 2018;23(6)

**DOI:** 10.2807/1560-7917.ES.2018.23.33.1808162

**Published:** 2018-08-16

**Authors:** Eurosurveillance editorial team

**Affiliations:** 1 European Centre for Disease Prevention and Control (ECDC), Stockholm, Sweden

Following expert consultation (Sally Partridge, Centre for Infectious Diseases and Microbiology, The Westmead Institute for Medical Research, The University of Sydney, Westmead Hospital, Australia; Daniel Haft, National Center for Biotechnology Information, National Institutes of Health, Maryland, United States) the *mcr* variant described in the article ‘Multiplex PCR for detection of plasmid-mediated colistin resistance determinants, mcr-1, mcr-2, mcr-3, mcr-4 andmcr-5 for surveillance purposes’ by Rebelo et al., as *mcr-4.3* (V236F) was updated to *mcr-4.6*.

The gene sequence was submitted to GenBank with the name *mcr-4.6* and the accession number is MH423812.

